# Functional Brain Networks: Does the Choice of Dependency Estimator and Binarization Method Matter?

**DOI:** 10.1038/srep29780

**Published:** 2016-07-15

**Authors:** Mahdi Jalili

**Affiliations:** 1 School of Engineering, RMIT University, Melbourne, Australia

## Abstract

The human brain can be modelled as a complex networked structure with brain regions as individual nodes and their anatomical/functional links as edges. Functional brain networks are constructed by first extracting weighted connectivity matrices, and then binarizing them to minimize the noise level. Different methods have been used to estimate the dependency values between the nodes and to obtain a binary network from a weighted connectivity matrix. In this work we study topological properties of EEG-based functional networks in Alzheimer’s Disease (AD). To estimate the connectivity strength between two time series, we use Pearson correlation, coherence, phase order parameter and synchronization likelihood. In order to binarize the weighted connectivity matrices, we use Minimum Spanning Tree (MST), Minimum Connected Component (MCC), uniform threshold and density-preserving methods. We find that the detected AD-related abnormalities highly depend on the methods used for dependency estimation and binarization. Topological properties of networks constructed using coherence method and MCC binarization show more significant differences between AD and healthy subjects than the other methods. These results might explain contradictory results reported in the literature for network properties specific to AD symptoms. The analysis method should be seriously taken into account in the interpretation of network-based analysis of brain signals.

Human brain is a complex network composed of regions connected through a networked structure. Recently, many research studies applied graph theory tools to signals recorded from the brain^1^. The brain networks can be studied in two categories: anatomical and functional. Anatomical brain networks are often extracted using Diffusion Tensor Imaging (DTI) technique^2^, while the functional networks can be extracted by analysis data recorded using Electroencephalography (EEG), Magnetocephalography (MEG) or functional Magnetic Resonance Imaging (fMIR) modalities^3^,^4^,^5^. In functional brain networks, the nodes (or vertices) are individual brain regions (e.g., EEG/MEG sensor locations or regions of interests in fMRI) and the edges are the functional links connecting the nodes.

There are various research studies linking the brain cognitive functioning to its network structure^6^. Research studies showed that brain networks, similar to many other real-world networks, have non-trivial topological features such as small-world-ness and hierarchy; see a review in^1^,^7^,^8^. Various brain disorders have been shown to disrupt statistical and dynamical properties of its network structure, such as Alzheimer’s Disease (AD)^4^, schizophrenia^9^, epilepsy^10^, early blindness^11^, Autism^12^ and Parkinson’s disease^13^. AD is the source of dementia in more than 50% of the cases, which is mainly caused by early deterioration of cerebral circuitry^14^. AD alters structural and functional brain connectivity. fMRI-based studies have shown low-frequency fluctuations in the functional connectivity of AD brains^15^,^16^, while the high-frequency (especially in alpha and beta bands) abnormalities have been frequently reported in various EEG studies^17^,^18^,^19^. Graph theory tools have been extensively applied to fMRI and EEG signals recorded from AD painters, and reported various aspects of abnormalities in the network measures. AD brains are often characterized by loss of small-worldness in their functional networks^20^,^21^. These networks also show decreased communication efficiency (i.e., increased average path length between brain regions)^4^,^22^,^23^, as well as decreased synchronizability^24^.

The first step in studying the functional brain network is to extract a connectivity matrix indicating the strength of interactions between the brain regions. This matrix is a weighted all-to-all connected matrix where the weights represent the connection strengths. The connectivity between two nodes is often estimated using some kind of statistical correlation (or dependence). Various linear and nonlinear methods have been used to obtain these connectivity matrices from EEG, MEG or fMIR signals. Pearson correlation coefficient, for instance, is a time-domain connectivity measure that is applied to filtered time series to obtain functional dependencies between two nodes^3^,^25^,^26^,^27^. Coherence is another linear dependency measure between two nodes, which has been applied to estimate functional connectivity matrices^12^,^28^,^29^. This measure obtains a value between 0–1 for each frequency. These values are integrated over the range of desired frequencies to obtain the dependency value for that frequency band. Another class of dependency measures are those to detect nonlinear correlations between the time series. Phase lag index and phase order parameter have been used to construct the weighted connectivity matrices by estimating the phase synchrony between the nodes^30^,^31^,^32^. To apply these measures, first appropriate methods are used to extract individual phases from the time series, and then the synchronization value is calculated between the phases. Another measure to detect nonlinear correlations is synchronization likelihood^33^, which measures the level of generalized synchronization between the time series. These measures have been widely applied to brain signals^34^,^35^.

The brain networks can be studied in either weighted or unweighted (binary) fashions. Often the weighted connectivity matrices are binarized to minimize the noise level and obtain more meaningful interpretation of the results. However, there is no standard way for binarization, and each method has its advantages and pitfalls. The simplest method is to use a uniform threshold, i.e., if the connectivity value between two nodes is larger than a certain threshold, they are connected through an undirected binary link^9^,^20^,^34^. It is not straight forward to determine the single threshold value, and thus one has to study the network properties for a range of threshold values. The main problem with this method is that the extracted networks have different densities (i.e., number of edges). Topological properties of networks significantly depend on their density, and comparing networks for the same threshold value can be biased to this effect. A solution can be to apply thresholds such that the final binary networks have the same density value (different threshold value for each network)^3^,^5^,^36^. In order to be insensitive to the threshold or density values, one can study the minimum spanning tree of the networks^24^,^32^. However, the minimum spanning tree of a network is highly sparse and many significant local connections are neglected. One can also study the minimum connected component, in which the local connections are preserved^37^.

Previous studies of functional brain networks in AD have used different methods for connectivity estimation and network binarization. There are also some contradictory conclusions across the studies. In this manuscript, we consider EEGs recorded from AD patients and healthy controls, and investigate to what extent the network properties depend on the choice of analysis method. By applying different methods on EEGs recorded from AD and control groups, we find that the results highly depend on the choice of method and in some cases opposite conclusions are drawn. Therefore, one should seriously take into account the effect of analysis method when interpreting a network-based study of brain signals.

## Methods

### Subjects and EEG recording

The EEGs of 16 newly diagnosed patients suffering from AD symptoms (Age: 69.1 ± 10.6) and 14 healthy controls (Age: 68 ± 11.2) were considered in this study. The subjects were recruited from the Memory Clinic of the Neurology Department (CHUV, Lausanne). The clinical diagnosis of probable AD symptoms was made according to the NINCDS–ADRDA criteria^38^, and cognitive functions were assessed with the Mini Mental State Examination (MMSE^39^). To confirm the absence of psychoactive drugs use and cognitive deficits, or diseases that may interfere with cognitive functions, MMSE of potential control subjects were also tested. The AD and control groups were not different in their age and educational level. AD patients showed significantly less MMSE scores than control subjects; AD MMSE: 21 ± 4.5, Controls’ MMSE: 29 ± 1 (P < 0.0001; Wilcoxon’s ranksum test). All the patients, caregivers, and control subjects gave written informed consent. All the applied procedures conform to the Declaration of Helsinki (1964) by the World Medical Association concerning human experimentation and were approved by the local Ethics Committee of Lausanne University.

The EEGs were recorded in resting-state condition with eyes-closed. The data were collected while subjects were sitting relaxed in a semi-dark room. To record the EEG data for duration of 3–4 minutes for each subject, the 128-channel Geodesic Sensor Net (EGI, USA) machine was used. The recordings were made with vertex reference at a sampling frequency of 500 Hz, and were further filtered (FIR, band-pass of 1–50 Hz; 50 Hz notch filter) and re-referenced against the common average reference. Then, the data were segmented into non-overlapping epochs each with 1-second length. All computations were first performed on individual epochs and then averaged over all artifact-free epochs in order to obtain the measures for each individual subject. Artifacts in all channels were edited off-line: first, automatically, based on an absolute voltage threshold (100 μV) and on a transition threshold (50 μV), and then on the basis of a thorough visual inspection. The sensors located in the outer ring showed low signal-to-noise ratio. They were further removed from the analysis, leaving 111 sensors. It is well-known that surface EEG is contaminated by volume conduction, which makes its interpretations limited. In order to minimize the effects of volume conduction (although not removing it completely), a high-resolution Laplacian transformation was used. For computing Laplacian transform of EEG signals, the CSD toolbox (psychophysiology.cpmc.columbia.edu/Software/CSDtoolbox) was used. In this work, we study the EEG signals only in alpha (7–13 Hz) band. A fifth-order Finite Impulse Response (FIR) filter was used to filter the original EEG time series. These subjects have been used in our previous studies for studying topography of synchronization maps and synchronizability of AD brains^18^,^24^,^40^.

### Computing connectivity matrices

The first step in studying functional brain networks is to compute connectivity matrices from EEG, MEG or fMRI time series. In this work we use EEG time series to obtain a 111-by-111 weighted connectivity matrix for each subject, where its entries show the strength of connectivity between the nodes (EEG sensor locations). We use four methods to compute the connectivity matrices: correlation, coherence, phase order and synchronization likelihood. Pearson correlation coefficient measures linear dependency in the time domain. The correlation coefficient between sensors *i* and *j* can be obtained as


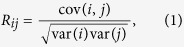


where cov(*i*,*j*) is the covariance between nodes *i* and *j*, and var(*i*) is the variance of node *i*. Coherence is another method to measure linear dependencies, which is computed in frequency domain. Coherence of sensors *i* and *j* in frequency *f* is calculated as


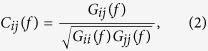


where *G*_*ij*_(*f*) is the cross-power spectral density at frequency *f* and *G*_*ii*_(*f*) is the auto-power spectral density. In order to obtain the coherence value for a frequency band, one should get the mean over the values for all frequencies of in that range.

Phase order and synchronization likelihood are based on different aspects of synchronization phenomena, both measuring nonlinear dependencies between the sensors. Two oscillators with phases *φ*_1_ and *φ*_2_ are called to be phase synchronized when the difference between their phase values are always less than a constant value. To compute phase synchrony between two time series, one needs to extract individual phases out of the filtered time series. Let’s suppose that the EEG time series of sensor *i* is 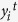, *t* = 1, …, *T*, where *t* indicates a sample in a single epoch and *T* is the number of available samples. Let’s consider the Hilbert transform of 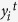 as 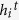
41. The instantaneous phase of the time series *y*_*i*_ is obtained as^42^


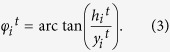


The degree of phase synchronization between sensors *u* and *v*, with phase values computed as above, is estimated by^42^


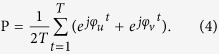


where *j* is the imaginary unit. This index scales as 0 ≤ *P* ≤ 1, where one has *P* ~ 0 for completely independent motion (uncoupled oscillators), and the case *P* ~ 1 indicates that the time series are phase synchronized.

Synchronization likelihood is another measure that quantifies nonlinear dependencies between two time series^33^. It is a measure of the generalized synchronization between two time series *y*_*i*_ and *y*_*j*_. The first step is to convert *y*_*i*_ and *y*_*j*_ as a series of state space vectors, which can be done using time delay embedding as


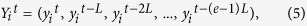


where *L* is the time lag and *e* is the embedding dimension. Synchronization likelihood measures the conditional likelihood that the distance between 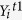 and 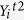 is smaller than *r*_*i*_ given that the distance between 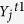 and 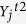 is smaller than *r*_*j*_. This value scales between 1 (for maximal synchronization) and a small non-zero value *P*_*ref*_ for independent motion. Let’s define correlation integral as


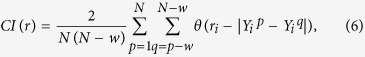


where *N* is the number of vectors, *w* is the Theiler correction for autocorrelation and *θ* is the Heaviside function. Then, one should choose *r*_*i*_ and *r*_*j*_ such that *CI*(*r*_*i*_) = *P*_*ref*_ and *CI*(*r*_*j*_) = *P*_*ref*_. The synchronization likelihood is defined as


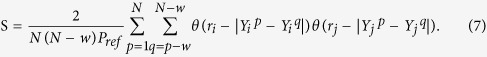


In this work, we set the parameters as *P*_*ref*_ = 0.01, *L* = 10, *e* = 10 and *w* = 0.1^34^.

### Constructing weighted and binary brain networks

The next step is to extract binary graphs from the weighted connectivity matrices. Not all the weighted links in the original connectivity matrices are significant, and one should use a method to remove the non-significant ones and minimize the noise level. Network binarization can be a good candidate solution to this problem; however, there is no unique strategy to binarize the connectivity matrices. In this work, we consider four methods to this end.

A simple method to binarize a weighted (often all-to-all connected) connectivity matrix is to apply a threshold *th*, that is if a link has a weight higher than *th*, the corresponding entry of the adjacency matrix is one, and zero otherwise. The problem with this method is that one cannot find a unique threshold value to extract only the significant links. The threshold values are often considered in a certain range and the^9^,^34^. Let’s denote this method by *Threshold*. There are individual variations in the functional connectivity; some subjects might have higher average functional connectivity than others. When one uses the same threshold for all subjects, the extracted networks will have different densities (i.e., number of links). Network density has a major role in many of its topological properties, and any observed pattern can be biased by this factor. In order to avoid this problem, one can study the network properties as a function of density instead of threshold^3^,^26^,^43^,^44^. The connectivity matrices are thresholded such that all extracted binary networks have the same density values. One can consider a range of density values and study topological properties of the extracted networks. Let’s denote this method by *Density*.

We also consider two other techniques to extract the binary networks: Minimum Spanning Tree (MST) and Minimum Connected Component (MCC). MST of a graph is defined as the subgraph that connects all nodes while minimizing the summation of link weights and without forming any loops. It has been shown that MST is insensitive to the threshold and density value, and can be considered as a good technique for graph binarization^32^. MST was first applied on brain networks in^45^, and then used by many studies, e.g., refs 24,31. The problem with MST method is that it results in a highly sparse network (a MST network with *N* nodes has *N*-1 edges), and thus many short-range connections (which is observed in many real systems) will be absent in MST. Another possible approach is to use MCC^37^, which is also a spanning subgraph and is constructed as follows. First, *N* nodes without any links are considered. Then, the strongest weight is considered and the corresponding binary link is created. Then, the second strongest weight is considered and so on. This procedure is continued until a connected graph is obtained, which is denoted as MCC. MCC is a spanning tree with at least *N*-1 edges and does not have the sparsity problem of MST.

### Graph theory metrics

As the binary functional networks are extracted, their topological properties are studied. There are many graph theory metrics in the literature; however not all of them are relevant for studying cognitive functions of the brain. Here we consider a number of neurobiologically relevant network measures. These metrics are related to brain cognitive functions including binding (information segregation and integration) and hierarchy. The first set of features is based on shortest paths between the nodes (global efficiency, nodes and edge betweenness centrality) that are related to the communicability of the brain and its integration properties. Global efficiency of a network is inversely proportional to its average shortest path length and is defined as^46^


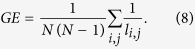


where *N* is the number of nodes and *l*_*ij*_ is the length of the shortest path between nodes *i* and *j*. High values of GE indicate efficient communication between the nodes.

In order to take into account the centrality of nodes/edges in the brain networks, their betweenness centrality^47^ is considered. Let’s denote the edge between nodes *i* and *j* by *e*_*ij*_. Edge-betweenness centrality *EBC*_*ij*_ of the network is defined by as


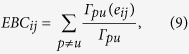


where Γ_*pu*_ is the number of shortest paths between nodes *p* and *u* in the graph and Γ_*pu*_(*e*_*ij*_) is the number of these shortest paths making use of the edge *e*_*ij*_. Node-betweenness centrality *NBC*_*i*_ is a centrality measure of node *i* in a graph, which shows the number of shortest paths making use of node *i* (except those between the *i*-th node with the other nodes)^47^. One can compute it as


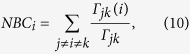


where Γ_*jk*_ is the number of shortest paths between nodes *j* and *k* and Γ_*jk*_(*i*) is the number of these shortest paths making use of the node *i*.

We consider local efficiency and modularity index as metrics characterizing hierarchal structure and segregation properties of the brain. Local efficiency is analogous to clustering coefficient (or transitivity), and is calculated as follow. Local efficiency of node *i* is computed as^46^


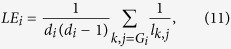


where *d*_*i*_ is degree of node *i* and *G*_*i*_ is the subgraph of neighbors of nodes *i* excluding node *i*. The local efficiency of the network is obtained by making average over all the nodes, more precisely


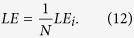


In order to capture the degree of modularity in the network with predetermined *M* modules, we use the following index^48^


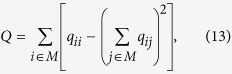


where the network is fully partitioned into *M* non-overlapping modules (clusters), and *q*_*ij*_ represents the proportion of all links connecting nodes in module *i* with those in module *j*. The modularity index is computed by estimating the optimal modular structure for a given network.

Networks may undergo random and/or intentional failures in their components, and their resiliency against such a failure is of high importance for their proper functioning. Degree-degree correlation has a significant role in determining resiliency of complex networks, which can be quantified by calculating the assortativity measure, as defined by^49^


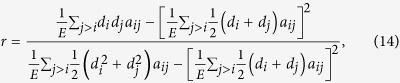


where *E* is the total number of the edges in the network. If *r* > 0, the network is assortative, whereas *r* < 0 indicates a disassortative network. For *r* = 0 there is no cor*r*elation between the node-degrees. In assortative networks, high-degree nodes often tend to interconnect, whereas in disassortative networks, nodes with high degree tend to connect to low-degree nodes. Assortative networks are likely to consist of mutually coupled hub nodes with high degrees and to be resilient against random failures. In contrast, the disassortative networks are likely to have vulnerable high-degree nodes^49^.

### Statistical assessments

In order to assess whether AD and control groups have statistically significant network properties, we use non-parametric Wilcoxon’s ranksum test. The results with *P* < 0.05 are considered to be statistically significant. All computations are performed using MatLab and its associated toolboxes.

## Results

### Global communicability

**Figure 1:**
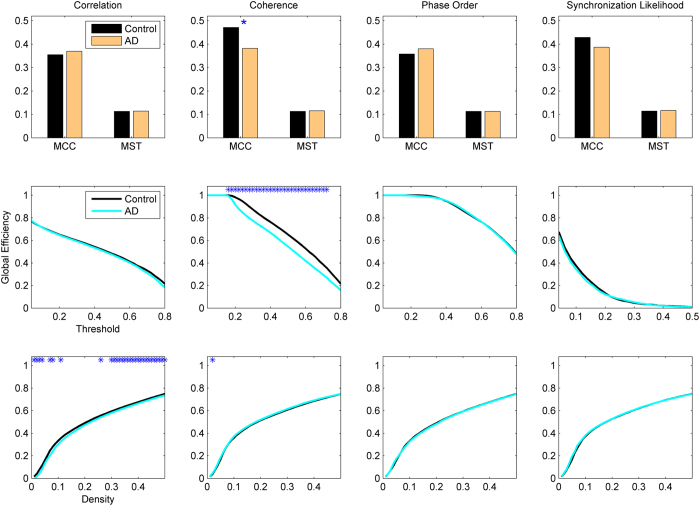
Global Efficiency of EEG-based functional networks in AD and healthy controls in alpha band (7–13 Hz). The graphs show group-level mean values with the asteric above the lines indicating a significant difference between AD and Control groups (P < 0.05; Wlcoxon’s ranksum test). Different methods are used to estimate the connectivity matrices, from left column to the right: Correlation, Coherence, Phase Order, and Synchronization Likelihood. The weighted connectivity matrices are binarized using MCC or MST (top panel), Threshold (middle panel), or Density (bottom panel); see the text for complete description of the methods.

**Figure 2:**
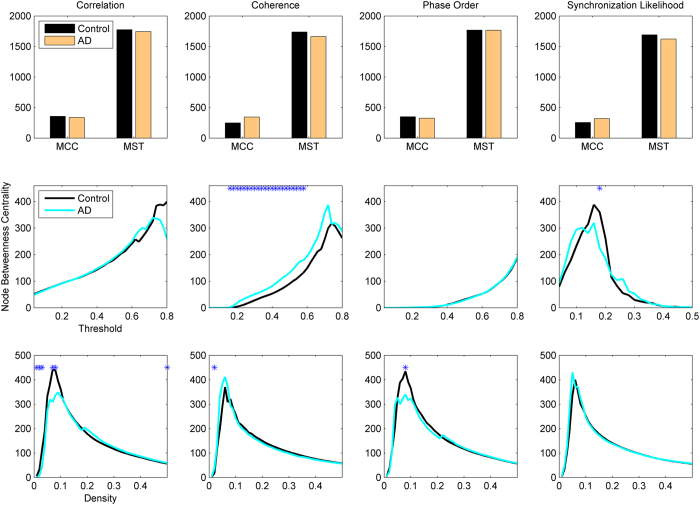
Node betweenness centrality in AD and healthy brain networks. Other designations are as Fig. 1.

**Figure 3:**
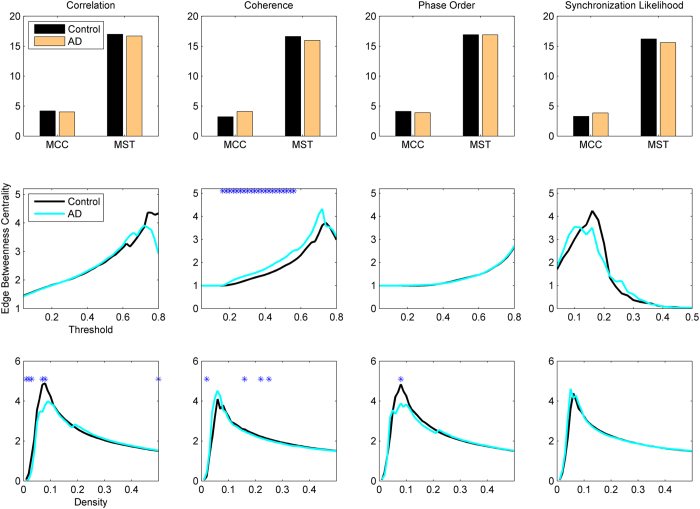
Edge betweenness centrality in AD and healthy brain networks. Other designations are as Fig. 1.

The first set of metrics studied in this work is those related to the global communicability between the nodes and include global efficiency, node and edge betweenness centrality, which are computed based on shortest paths. Fig. 1 shows the global efficiency when different methods are used. There are variations across the connectivity extraction and binarization methods. The nonlinear methods (phase order and synchronization likelihood) show significant differences between AD and controls in neither of binarization methods. When the connectivity values are estimated by coherence, AD networks show significant decrease in the global efficiency when MCC or Threshold are used for binarization. This pattern is observed for Density when correlation is used for connectivity estimation. We find similar pattern for the node and edge betweenness centrality measures (Figs 2 and 3). We find extensive increase of node/edge betweenness centrality in AD brains for different threshold values when coherence is used to measure the connectives. Apart from some patchy changes (both decrease and increase), there are no significant changes for the other connectivity estimation methods.

### Local connectivity

**Figure 4:**
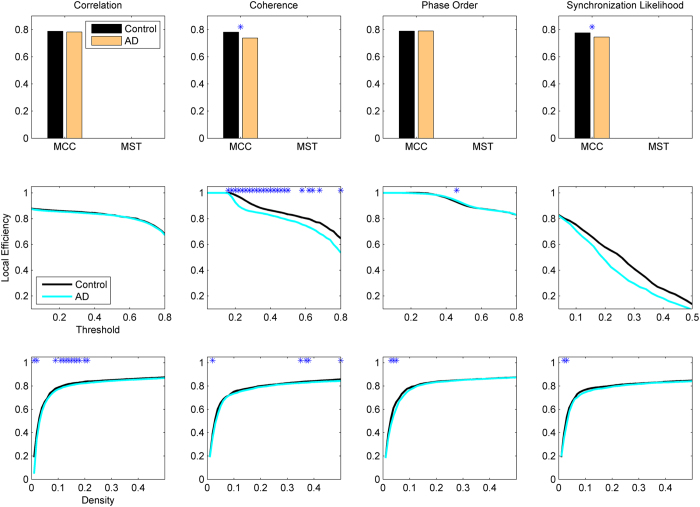
Local efficiency in AD and healthy brain network. Other designations are as Fig. 1.

**Figure 5:**
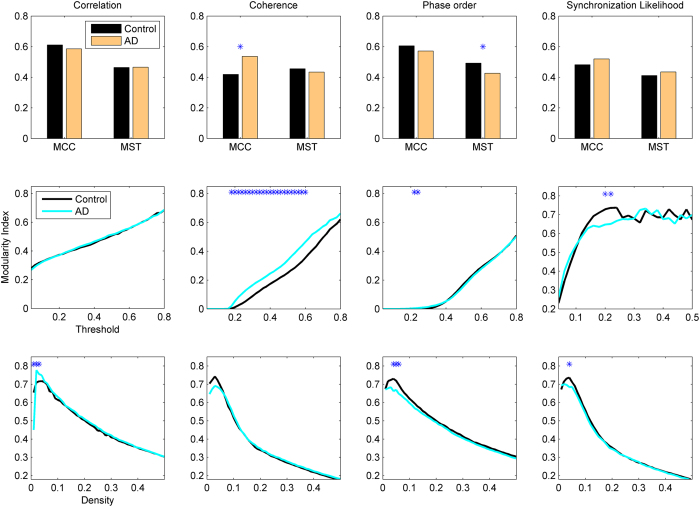
Modularity index in AD and healthy brain networks. Other designations are as Fig. 1.

The local efficiency and modularity index are used to measure the strength of local connections and hierarchical structure, and the results are shown in Figs 4 and 5, respectively. AD networks show significant decline of the local efficiency when coherence or synchronization likelihood is used for connectivity estimation and MCC for binarization. Note that MST results in the minimum spanning tree for which the local efficiency for each node is zero. MST extracts the minimum spanning three, and thus when a node is removed from its neighbouring set, they will be disconnected with zero local efficiency. Likewise, clustering coefficient will also be zero in the networks extracted by MST. Thus, MST cannot capture the profile of local connectivity, while other metrics do not have this problem. Choosing Threshold as the binarization method, coherence estimation shows significant decrease of the local efficiency for a broad range of the threshold values, while other methods do not show any changes. All connectivity extraction methods show decreased local efficiency for a range of small density values. The patterns for the modularity index are inconsistent; MCC/coherence shows significant increase of modularity in AD networks, whereas MST/phase order shows decrease of the modularity in AD. Similar to other metrics, the modularity index also shows significant changes in AD brains for a broad range of threshold values in Threshold/coherence, while there are only some patchy changes for other methods.

### Degree-degree correlation

**Figure 6:**
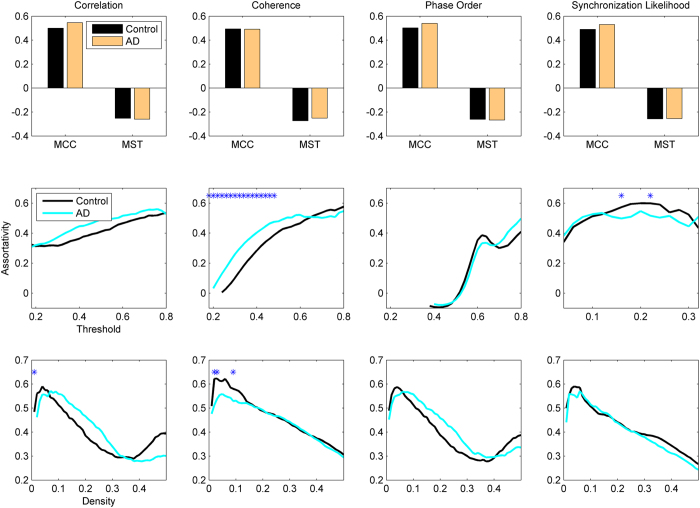
Assortativity in AD and healthy brain networks. Other designations are as Fig. 1.

Figure 6 shows the assortativity values for different methods. MCC and MST show completely opposite patterns. While both AD and control brains are assortative under MCC binarization, they are disassortative under MST binarization method. However, there are significant differences for AD vs. controls in neither of them. AD networks show significantly higher assortativity than healthy brains for Threshold/coherence, while other methods do not identify significant differences apart from few threshold or density values.

## Discussion and Conclusion

Tools available in graph theory and network science have been extensively applied to study the human brain. Studying network properties in various brain disorders have revealed their abnormal behaviour. Previous studies showed that functional and anatomical networks in AD patients demonstrate altered network properties such as average path length, clustering coefficient, small-worldness and synchronizability^4^,^24^,^50^,^51^,^52^. These studies often use a dependency estimation method such as correlation, coherence, phase order index and synchronization likelihood. Although these methods quantify the strength of connectivity between two nodes, they measure different aspects of functional connectivity. Cross-correlation is a way to decide the extent to which two nodes covary, while coherence indicates “similarity” by looking at the similarity for two nodes in frequency space, rather than time space. It has been shown that coherence values are smaller than correlation values for a signal with any noise, however, coherence is more robust for increasing level of noise^53^. Correlation and coherence measure linear dependencies between two time series, and cannot capture nonlinear interconnections. Synchronization likelihood and phase order index can detect the effects caused by nonlinear connectivity. Synchronization likelihood measures the generalized synchronization that is to what extent one of the variables is synchronized with a general function of the other variable. Phase order index computes the synchronization in the phase space. All these measures are sensitive to the volume conduction effect (although phase order index being less sensitive than others), and proper pre-processing should be applied (e.g, Laplacian or source transformation) to minimize the unwanted effects of volume conduction.

In this work we studied EEG-based complex network properties of functional brain networks in AD. EEG, as a non-invasive and cheap neuroimaging modality, has been extensively used to study AD mechanisms in the brain^18^,^54^,^55^,^56^. To study AD-specific properties of functional brain networks, we applied different connectivity estimation and binarization methods on EEGs recorded from AD patients and healthy control subjects. The literature on network properties of AD brain is contradictory, which is partially due to the use of different analysis methods. Table 1 summarizes previous research studies on the functional network properties of AD brains. These studies used various neuroimaging modalities including fMRI, PET, EEG and MEG. Each study used one of the four dependency estimation methods considered in this work, and Density, Threshold or MST as binarization method. There was no study using MCC and some of them did not indicate the binarization method.

Previous studies reported contradictory findings. For example, while Zhao *et al*.^52^ and Afshari *et al*.^22^ reported increase of local efficiency, others reported decrease of local connectedness measured by clustering coefficient^20^,^57^,^58^,^59^,^60^,^61^. Some reports did not find any significant changes in the clustering coefficient^4^,^62^. Note that local efficiency is analogous to clustering coefficient, both measuring local connectedness among the nodes. Our data showed either decreased or no change in the local efficiency of AD brains, depending on the connectivity estimation and binarization method used for the analysis. This is consistent with many of the previous reports. Therefore, the local connections are likely to be destroyed in AD, which is captured by both types of connectivity estimation methods, linear (coherence and correlation) and nonlinear (phase order and synchronization likelihood), although by different confidence levels.

Global efficiency, which measures the global communicability in the network is inversely proportional to the average path length, i.e., the higher is the average path length, the lower the global efficiency. Previous studies reported contradictory findings for the average path length (or global efficiency) as well. Studies carried out by Wang *et al*.^23^, Li *et al*.^61^, Zhao *et al*.^52^, Afshari *et al*.^22^ and Stam *et al*.^4^ reported increase of average path length (decrease of global efficiency) in AD brains, while De Haan *et al*.^58^ and Sanz-Arigita *et al*.^62^ reported decrease of average path length in AD indicating better global communicability of AD brain than healthy brains. Some studied did not find any AD-specific changes in the average path length. Depending on the connectivity estimation and binarization method, we found either decreased or no AD-specific changes in the global efficiency, which is not in agreement with^58^,^62^. These two reports used synchronization likelihood to estimate the weighted connectivity matrices, for which we did not identify any significant differences between AD and healthy brains. Previous works did not report the node and edge betweenness centrality for AD brains. We found that the AD-specific changes in the betweenness values highly depend on the analysis method. While coherence showed increase of betweenness in AD brains (wide-spread change for Threshold and some patchy changes for Density), other methods showed either no change or decreased betweenness in AD.

Note that none of methods are right or wrong per se and each has its own interpretation. Therefore, one should take into account the analysis method when interpreting results obtained by applying network theory tools to the brain.

## Additional Information

**How to cite this article**: Jalili, M. Functional Brain Networks: Does the Choice of Dependency Estimator and Binarization Method Matter? *Sci. Rep.* **6**, 29780; doi: 10.1038/srep29780 (2016).

## Figures and Tables

**Table 1:** Previous studies reporting abnormalities of functional brain networks in AD.

Study	Brain signal	Connectivity measure	Binarization	Findings
Seo, *et al*.^57^	PET	Correlation	Density	Decreased CC and no change in APL
van Haan *et al*.^63^	MEG	Synch Likelihood	NA	Decreased modularity in lower bands and increased modularity in higher bands
Stam *et al*.^4^	EEG	Synch Likelihood	Threshold/Density	Increased APL and no change in CC
De Haan & Jalili^58^	EEG	Synch Likelihood	Density	Decreased CC in alpha and beta bands and decreased APL in alpha and gamma bands
Afshari *et al*.^22^	EEG	Coherence	Density	Decreased GE and increased LE in alpha and beta bands
Spekar *et al*.^59^	fMRI	Correlation	Threshold	Decreased CC
Zhao *et al*.^52^	fMRI	Correlation	Density	Increased LE and decreased GE
Ciftci^64^	fMRI	Coherence	MST	No change in the degree distribution
Sanz-Arigita *et al*.^62^	fMRI	Synch Likelihood	Threshold/Density	Decreased APL and no change in CC
Stam *et al*.^20^	MEG	Phase Synch	NA	Decreased CC and APL in alpha band
Brier *et al*.^60^	fMRI	Correlation	Density	No change in APL and reduced CC and modularity
Li *et al*.^61^	fMRI	Correlation	Threshold	Decreased GE and CC
Wang *et al*.^23^	fMRI	Correlation	Threshold	Increased APL

APL: Average Path Length, CC: Clustering Coefficient, GE: Global Efficiency, LE: Local Efficiency.
